# One-Day Two-Fraction Radiosurgery for Brain Metastases Using Gamma Knife

**DOI:** 10.7759/cureus.6026

**Published:** 2019-10-29

**Authors:** Yoshimasa Mori, Yoshihisa Kida, Yasuhiro Matsushita, Ryota Nishimura, Kazuki Kusu, Atsuo Masago

**Affiliations:** 1 Radiation Oncology and Neurosurgery, Center for Advanced Image-guided Radiation Therapy, Shin-Yurigaoka General Hospital, Kawasaki, JPN; 2 Neurosurgery, Ookuma Hospital, Nagoya, JPN; 3 Neurosurgery, Gamma Knife Center, Ookuma Hospital, Nagoya, JPN

**Keywords:** radiosurgery, gamma knife, hypofraction, brain, tumor, metastasis, fractionation

## Abstract

Objective: We aimed to evaluate the feasibility of a one-day two-fraction Gamma Knife radiosurgery (GKRS) for brain metastases.

Cases and methods: Ten cases with ten brain metastases (four cases of lung adenocarcinoma, one small cell lung carcinoma (SCLC), two renal cell carcinoma, one breast cancer, one esophageal carcinoma, and one bile duct carcinoma) were treated by one-day two-fraction (with an interval of more than six hours) GKRS under rigid skull frame fixation. Of the ten brain metastases, five lesions were in the frontal lobe, one in temporal, one in occipital, and three in the cerebellar hemisphere. The mean planning target volume (PTV) of the ten brain tumors was 7.8 ml (median, 8.0; range, 3.8 - 11.8). The ten targets of the mean prescription isodose volume (PIV) of 10.1 ml (median, 10.1; range, 4.4 - 15.9) were treated with a mean margin dose of 20.4 Gy (median, 20.5; range, 16.4 - 22) in two fractions. In five cases, other small brain metastases (one to seven tumors) were also treated simultaneously in a single fraction GKRS. The indication of two-fraction radiosurgery was large lesion size in eight, retreatment in three, the proximity of the motor area in three, and pre-existing perifocal edema symptom of dysarthria in two, nausea and vomiting in one, and dementia in one.

Results: Eight cases were alive at the end of the follow-up period of one to nine months (median, 6). One patient with SCLC died four and a half months after GKRS, from aggressive regrowth of the treated frontal lesion after transient marked shrinkage. Another patient died four months after GKRS due to the progression of other brain tumors treated by single fraction GKRS at the same time. In nine of 10 cases, the size of the treated tumors was controlled until the end of the follow-up period or the patient’s death. In two cases, an additional GKRS was performed for newly developed brain metastases at distant locations at six months and five months after one-day two-fraction GKRS, respectively, and controlled at the end of the follow-up period.

Conclusions: A relatively high dose may be safely delivered to large lesions, to those close to the important structures, or those with perifocal edema by one-day two-fraction radiosurgery. Local control was good except for a relapsed SCLC metastasis case. Evaluation in more cases with a longer follow-up period is necessary to determine definite indications and optimal prescription doses.

## Introduction

The effectiveness of stereotactic radiosurgery (SRS) has been reported in the treatment of various brain disorders [1-4]. However, single-fraction SRS has traditionally been limited to small lesions, usually up to 3 to 4 cm in diameter, because of the increased risk of radionecrosis in surrounding normal brain with increasing volume treated [5-8]. In addition, even if the size of the lesions is 2 to 4 cm, the single fraction irradiation dose would be decreased so as not to increase the possibility of adverse effects on the surrounding normal brain [5,9,10]. Staged- or hypofractionated-SRS regimens have emerged as an alternative to single-fraction treatment when the target tumors are large [6,7,11-17]. Staged- or hypofractionated SRS maintains the stereotactic advantages of precise tumor localization and sharp dose fall-off while using the biologic advantage of fractionation, and might, therefore, improve the therapeutic ratio in selected patients [11]. Some reports on staged- and hypofractionated-SRS have been published [6,12,13,18-20]. However, there are limited data available indicating which dose and fractionation scheme should be used, particularly when treating large brain metastases [11].

With the development of noninvasive stereotactic methods, hypofractionated SRS regimens can be applied more easily, even with Gamma Knife. Non-invasive head fixation systems with Extend (Elekta, Tokyo) and Icon (Elekta, Tokyo) are recently available. Perfexion (PFX), a recent version of Gamma Knife (GK), has enabled SRS using the Extend system, a rigid repositioning frame using a mouth-piece system [14,21]. In addition, a thermoplastic headshell is available in the latest generation Icon under the latest generation Icon system equipped with cone-beam computed tomography (CT) [22].

In this study, the feasibility of one-day two-fraction radiosurgery using Gamma Knife (GKRS) under skull frame fixation with pins was evaluated. This has the merit of precise targeting with rigid fixation using a skull frame and provides the advantage of fractionation in a short term treatment when single-session GKRS seemed to have a risk in the case of relatively large target lesions or some other situations.

## Materials and methods

The research ethics boards of Shin-Yurigaoka General Hospital and Ookuma Hospital approved the study. The need for patient consent was waived.

Ten cases with ten brain metastases from lung adenocarcinoma (four cases), small cell lung carcinoma (SCLC) (one case), renal cell carcinoma (two case), breast cancer (one case), esophageal carcinoma (one case), and bile duct carcinoma (one case) were treated by one-day two-fraction (with an interval more than six hours) GKRS under rigid skull frame fixation from May 2018 to February 2019 in the Gamma Knife Center, Ookuma Hospital (Table 1).

**Table 1:** Cases of brain metastases treated by one-day two-fraction Gamma Knife radiosurgery. GKRS=Gamma Knife stereotactic radiosurgery, PTV=planning target volume, PIV=prescribtion isodose volume, fx.=fraction, Max.=maximum, SCLC=small cell lung carcinoma, PCI=prophylactic cranial irradiation, lt.=left, rt.=right, ca.=carcinoma, cbll=cerebellar, front.=frontal, occipit.=occipital, temp.=temporal, mos=months Case 3 had received 1st GKRS 6 months before. Case 8 had received 1st GKRS, a resection, and a 2nd GKRS before.

Case	Age/sex	Diagnosis of primary ca.	Location of brain tumor	Prior GKRS incl. other location	Prior resection	Retreatment	PTV volume (ml)	PIV volume (ml)	Total marginal dose (Gy)	Max. total dose (Gy)	Other targets
1	67/F	breast	lt.cbll	no	no	initial	7.1	8.7	22 Gy/ 2 fx.	44 Gy/ 2 fx.	yes (6 more)
2	66/F	lung (adenoca.)	lt.front	3 times	no	yes (9 mos. 4 mos.)	7.9	9.8	19 Gy/ 2 fx.	38 Gy/ 2 fx.	none
3	63/F	lung (SCLC)	rt.cbll	2 times	no (PCI 12 mos.)	yes (6 mos)	4.9	10.7	16.4 Gy/ 2 fx.	32.8 Gy/ 2 fx.	yes (4 more)
4	69/F	lung (adenoca.)	rt.front	no	no	initial	11.8	15.9	20 Gy/ 2 fx.	40.4 Gy/ 2 fx.	yes (7 more)
5	75/M	lung (adenoca.)	rt.occipit	1 time	no	initial	8.1	11.1	20 Gy/ 2 fx.	40 Gy/ 2 fx.	none
6	66/M	lung (adenoca.)	rt.front	no	no	initial	11.2	12.9	20.4 Gy/ 2 fx.	40.8 Gy/ 2 fx.	yes (1 more)
7	72/M	kidney	rt.cbll	no	no	initial	3.8	4.4	22 Gy/ 2 fx.	44 Gy/ 2 fx.	none
8	67/M	esophagus	lt.temp	2 times	yes (7 mos)	yes (23 mos, 3 mos)	6.2	8.8	20.6 Gy/ 2 fx.	41.2 Gy/ 2 fx.	none
9	58/M	kidney	lt.front	no	no	initial	8.6	10.4	22 Gy/ 2 fx.	44 Gy/ 2 fx.	yes (1 more)
10	85/M	bile duct	lt.front	no	no	initial	8.1	8.7	22 Gy/ 2 fx.	44 Gy/ 2 fx.	none
mean							7.8	10.1	20.4 Gy/ 2 fx.	40.9 Gy/ 2 fx.	
median							8	10.1	20.5 Gy/ 2 fx.	41 Gy/ 2 fx.	

In four of the ten cases, GKRS was done for recurrent brain metastases after prior GKRS sessions (one to three times). In three of them, a local or marginal recurrent brain tumor was treated (Case 3, in the second procedure; Cases 2 and 8, in the third). The ten brain metastases were located in the frontal lobe (five lesions), temporal (one), occipital (one), and cerebellar hemisphere (three). The mean planning target volume (PTV) in the ten cases was 7.8 ml (median, 8.0; range, 3.8 - 11.8). The ten brain tumors of the mean prescription isodose volume (PIV) of 10.1 ml (median, 10.1; range, 4.4 - 15.9) were treated with the mean margin dose of 20.4 (median, 20.5; range, 16.4 - 22) Gy in two fractions. GKRS plan was made on a GammaPlan workstation (Elekta, Tokyo) in a series of stereotactic CT images using the Leksell skull frame (Elekta, Tokyo) with quick fixation skull screws. Magnetic resonance images (MRI) with Gadolinium-enhancement, taken prior to the procedures mostly on the preceding day, were co-registered on the CT images and were used for tumor delineation. Two-fraction irradiation, with an interval of more than six hours between them, in the morning and the afternoon, was performed with Leksell GammaKnife Perfexion (Leksell, Tokyo). The skull frame was kept on the patient head from before the scanning of stereotactic CT until the completion of the second fraction irradiation. In five cases, other small brain metastases (one to seven tumors) were also treated simultaneously in a single fraction GKRS. Steroids were administered orally before and after irradiation in Cases 2, 6, 7, 9, and 10. It was continued in Case 2 and 9 and then tapered off. In Case 3, oral steroid was given for one month after GKRS to relieve perifocal brain edema.

The indication of two-fraction radiosurgery was large lesion size in eight cases (Cases 1, 2, 4, 5, 6, 8, 9, and 10), retreatment in three (Cases 2, 3, and 8), proximity of the motor area in three (Cases 4, 7, and 10), and pre-existing perifocal edema with symptom of dysarthria in two (Cases 4 and 10), nausea and vomiting in one (Case 7), and apathetic mental condition in one (Case 9).

The follow-up of the cases was based on both clinical status and imaging results every one or two months.

## Results

Eight cases were alive at the end of the follow-up period (median, 6 months; range, 1-9 months). One patient with SCLC (Case 2) died, four and a half months after GKRS, from aggressive regrowth of the treated frontal lesion after transient marked shrinkage. Another patient (Case 3) died four months after GKRS due to the progression of other brain tumors treated by single fraction radiosurgery at the same time. In nine of 10 cases, other than Case 2, the size of the treated tumors was controlled (Table 2).

**Table 2:** Treatment results of one-day two-fraction Gamma Knife radiosurgery. FU=follow-up; CR=complete response (i.e., the disappearance of the treated tumor); PR=partial response (i.e., tumor shrinkage, more than half); NC=no change; PG=progression; GKRS=Gamma Knife stereotactic radiosurgery; Cbll=cerebellar

Case	FU (mos.)	Additional GKRS sessions for other new lesions	Local results	Survival	Adverse effects	Remark
1	1	no	PR	alive	none	
2	4.5	no	PG	dead	none	
3	4	no	PR	dead	none	
4	9	no	PR	alive	none	Preexisting nausea due to another cbll lesion was relieved.
5	7	7 lesions (6 mos)	CR	alive	none	
6	9	no	NC	alive	deterioration of perifocal edema	No symptoms under administration of steroid
7	8	no	PR	alive	none	
8	7	3 lesions (5 mos)	PR	alive	none	
9	4	no	PR	alive	none	
10	5	no	CR	alive	none	
mean	5.9					
median	6					

In one case (Case 6), pre-existing perifocal edema worsened after treatment, though the size of the treated tumor was unchanged. However, the clinical symptoms were stable with oral administration of steroid. No other acute or subacute symptomatic adverse effects were observed associated with the treatment in the other nine cases. In Case 4, pre-existing nausea due to a cerebellar lesion other than the main lesion treated by one-day two-fraction GKRS was relieved after single-fraction GKRS at the same time. Pre-existing symptoms due to perifocal edema of the treated tumor disappeared or improved somewhat in Case 4, 7, 9, and 10. In Cases 5 and 8, additional GKRS was performed for newly developed small brain lesions in distant locations at six months and five months after one-day two-fraction GKRS, respectively. All those tumors were controlled at the end of the follow-up period.

**Figure 1:**
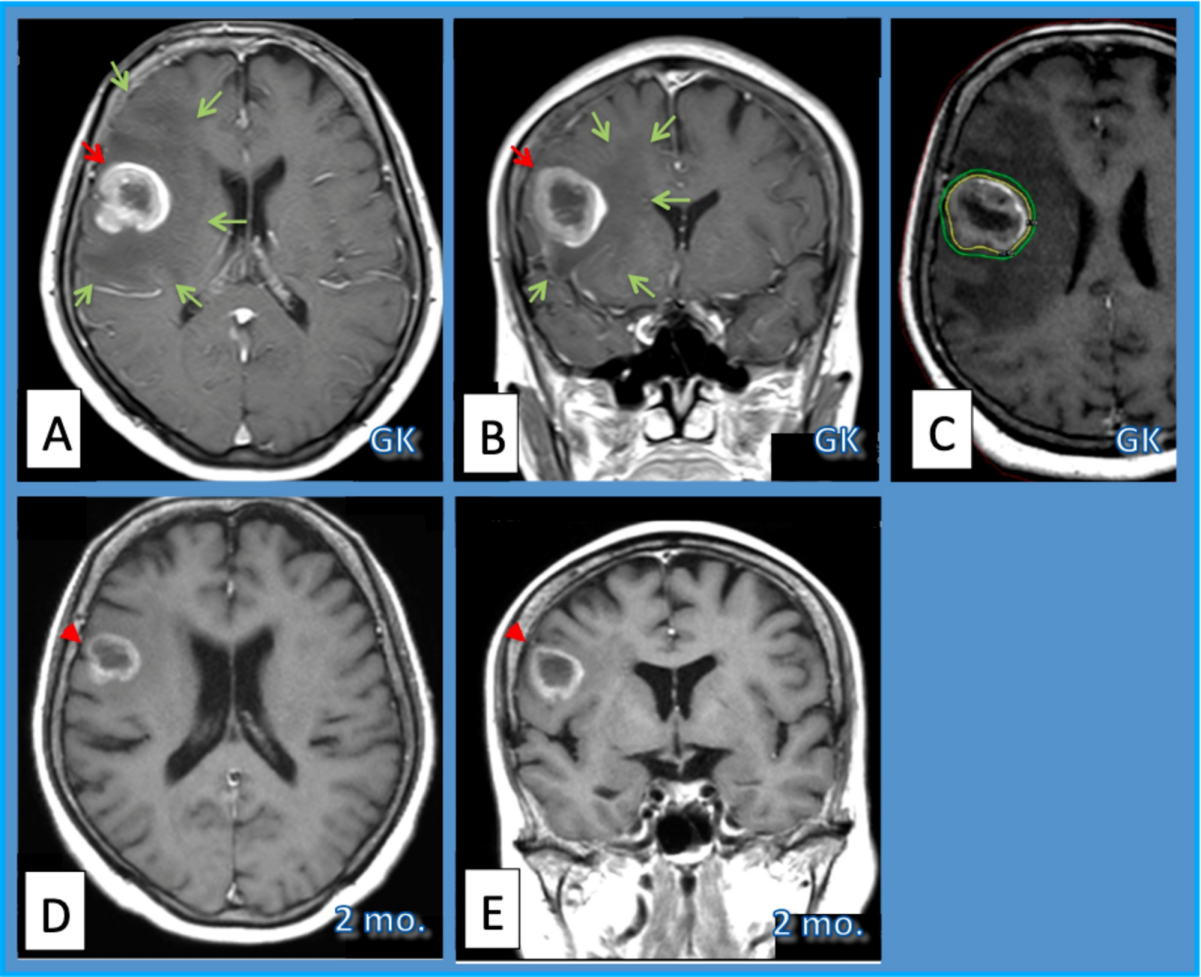
Case 4. Case 4.  Sixty-nine-year-old woman with lung adenocarcinoma brain metastasis. Axial (A) and coronal (B) magnetic resonance images (MRIs) with gadolinium (Gd) enhancement from before Gamma Knife radiosurgery (GKRS) show a right frontal mass lesion (red arrows). A margin dose of 20.4 Gy in 2 fractions (fx.) was delivered for a prescription isodose volume (PIV) of 15.9 ml on GammaPlan (Elekta, Tokyo) workstation (C). The yellow line shows prescription isodose and the green line indicates the isodose line of 16 Gy/ 2 fx. The lesion shrank somewhat (red arrowheads) and the peritumoral edematous area of low-intensity (green arrows) became less wide within two months after one-day two fraction GKRS (D: axial, E: coronal MRI).

**Figure 2:**
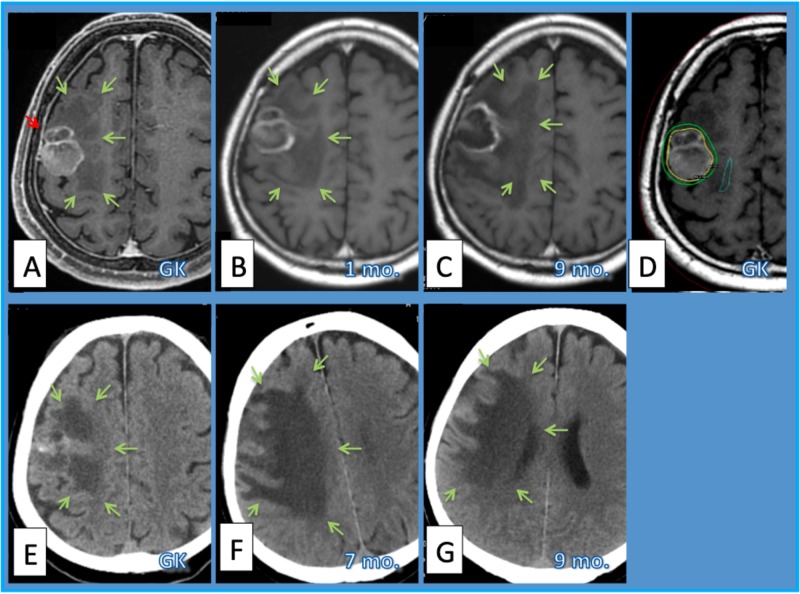
Case 6. Case 6.  Sixty-six-year-old man with lung adenocarcinoma brain metastasis.  Gd-enhanced MRI before GK (A) showed a right frontal mass lesion (red arrow) causing perifocal edema of low-intensity (green arrows). The volume of the tumor had been stable in MRI one month after GKRS (B) and in nine months after GKRS (C).  The wall of the cystic lesion became thinner within nine months after GKRS. The perifocal edema showed a peak on MRI seven months after GKRS (F) and was a little improved on CT nine months after GKRS (G). E: Plain CT before GKRS. Dose planning of GKRS (margin dose=20 Gy/ 2fx, PIV=12.9 ml) on GammaPlan (D). The yellow line shows the prescription isodose and the green line is the isodose line of 16 Gy/ 2 fx. One month after GKRS (C) MRI showed decreased central enhancement inside it, but the edematous area spread wider. Two months after GKRS enhanced volume became much smaller on iodine-enhanced computed tomography (D) and the edematous area improved a little. MRI = magnetic resonance imaging, GK=Gamma Knife, GKRS = GK radiosurgery, fx. = fraction

**Figure 3:**
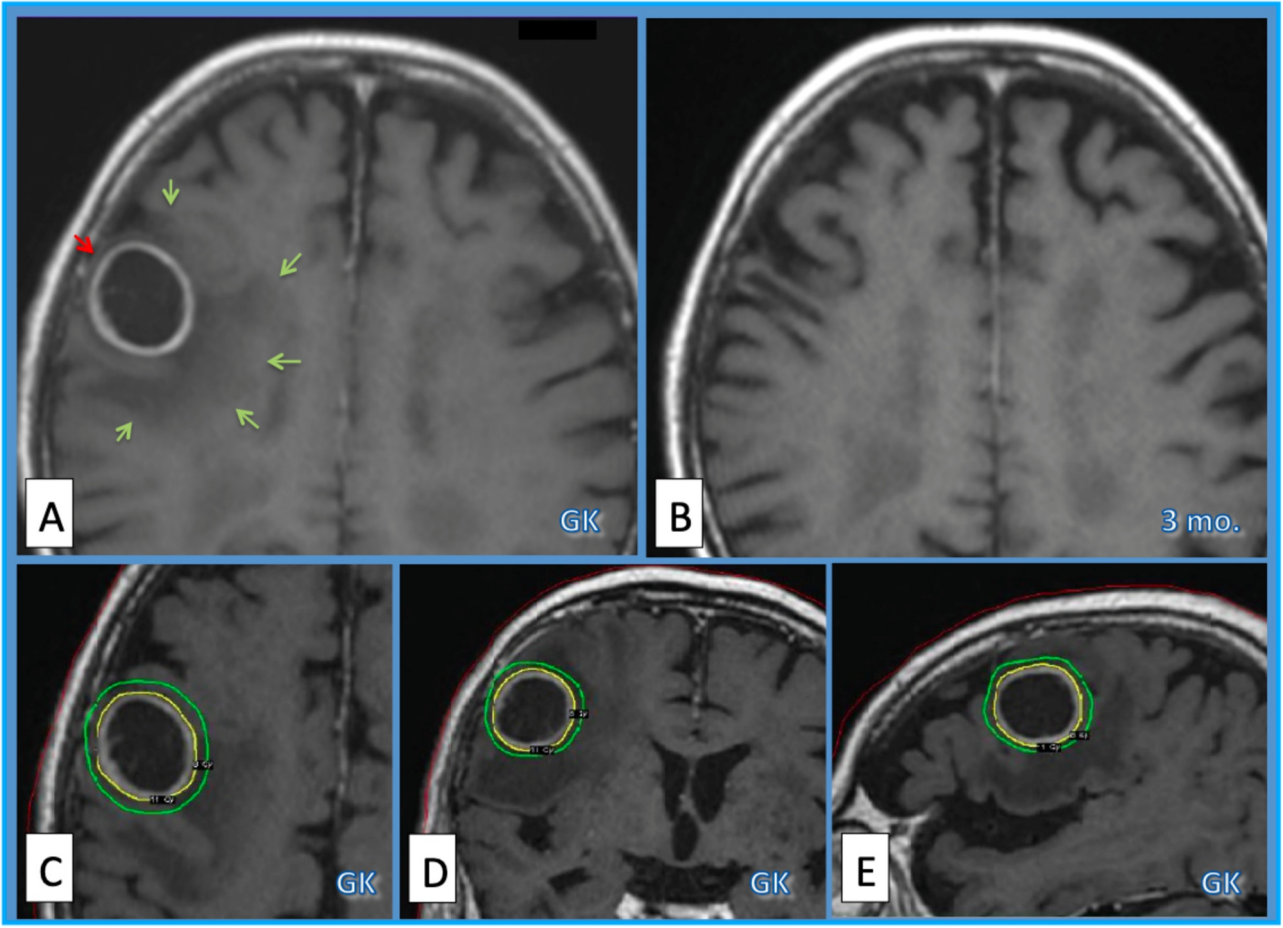
Case 10. Case 10.  Eighty-five-year-old man with bile duct carcinoma brain metastasis.  Gd-enhanced MRI before GKRS (A) showed a right frontal mass lesion (red arrow) causing perifocal edema of low-intensity. Three months after GKRS (B) the lesion disappeared and the perifocal edema (green arrows) improved completely. Dose planning of GKRS (margin dose=20 Gy/ 2fx., PIV=15.9 ml) on GammaPlan (C, axial; D; coronal; E, sagittal). The yellow line shows prescription isodose, and green line is the isodose line of 16 Gy/ 2 fx. MRI = magnetic resonance imaging, Gd = gadolinium, GKRS = Gamma Knife radiosurgery, PIV = prescription isodose volume, fx. = fraction

Three representative cases (Cases 4, 6, and 10) are shown in Figures 1-3.

## Discussion

SRS is thought to be an effective treatment option for brain lesions including metastatic brain tumors, if the target is not large [1]. If the target is large, we use a lower prescription dose to reduce the risk of radiation injury in the surrounding normal brain, because the target volume and the risk of permanent symptomatic brain injury have been shown to be correlated [5,9,10]. Kondziolka et al. provided a graph displaying tumor (meningioma) volume and marginal dose indicating cases developing post-GKRS adverse effect [9]. The graph was traditionally an important reference when we selected the marginal dose. Minniti et al. used reduced doses associated with larger tumor volume in a linear accelerator (LINAC)-based single session-SRS [5]. Radiosurgical dose was 20 Gy for metastases with a volume <4.3 cu cm (corresponding to a sphere of 2 cm in diameter), 18 Gy for metastases with a volume of 4.3-14.1 cu cm, and 15-16 Gy for metastases with a volume of >14.1 cu cm or located in the brainstem. Shaw et al. determined the maximum tolerated doses of single-fraction SRS for their patient population to be 24 Gy, 18 Gy, and 15 Gy for tumors <20 mm, 21-30 mm, and 31-40 mm in maximum diameter respectively [23]. Some reports noted a correlation between post-treatment brain radiation injury and the volume that received a specific dose, including surrounding structures other than the treated volume. Kano et al. reported a correlation of 12 Gy-volume (the volume of tissue, including target, receiving >12 Gy) during GKRS for brain arteriovenous malformation (AVM) [24]. Korytko et al. also reported a correlation of 12 Gy Volume and post-radiosurgical radionecrosis on imaging during GKRS for non-AVM intracranial tumors, including brain metastasis cases (53.5%= 92 / 198 cases) [25]. Blonigen et al. reported that 8 Gy- to 16 Gy- volumes are a useful predictor for post-treatment radionecrosis in brain metastasis cases treated by linear accelerator-based SRS, though most of their patients had received whole-brain radiation therapy previously [10]. They proposed that cases with 10 Gy-volume >10.5 cu cm or 12 Gy-volume >7.9 cu cm be considered for hypofractionated rather than single-fraction treatment, to minimize the risk of symptomatic radionecrosis. Minniti et al. described that 10 Gy-volume and 12 Gy-volume were the most predictive risk factors [5]. For 10 Gy-volume >12.6 cu cm and 12 Gy-volume >10.9 cu cm, the risk of radionecrosis was 47%.

Other than single-fraction SRS, staged-treatment and fractionated irradiation are also available to reduce the risk of perifocal radiation necrosis [6,7,11-13,15-18,26]. Fractionated doses help to spare normal tissues by allowing time for the repair of sublethal damage and/or potential lethal damage and enhance tumor cell killing by allowing reoxygenation between doses [27]. Konefal et al. reported that potential lethal damage recovery was nearly complete in a six-hour interval, and sublethal damage recovery was complete by two hours with a dose schedule of 8 Gy/ 2 fx. in an experimental study using normal fibroblasts and fibrosarcoma cells [27]. Under in vivo conditions with blood flow, reoxygenation is expected to enhance the effectiveness of irradiation to the target tumor [28]. The optimal dose fractionation for a large brain metastasis not responsive to single-fraction SRS has not been established well [15]. Inoue et al. reported the importance of a 14 Gy equivalent dose-volume in five-fraction CyberKnife SRS for large brain metastases [16]. A review by Masucci summarized the current (largely retrospective) data regarding outcomes after hypofractionated radiation therapy and staged SRS for large brain metastases from existing series [17]. Hypofractionated doses ranged from 22 to 42 Gy in three to five fractions, with one-year local control rates ranging from 56% to 100% and with prescribed doses often dependent on tumor volume or diameter. Kim et al. reported the treatment results of hypofractionated GKRS [7]. The mean gross tumor volume was 18.3 ml. The median dose was 80 Gy (at 50 Gy isodose line) with three fractions for three consecutive days (range, 5-11 Gy, and 2-4 fractions for 2-4 consecutive days). The local control rate was 90%. Radiation necrosis developed only in 2.7% (1 /36) of cases. As an alternative to daily hypofractionated SRS, staged SRS is a reasonable option. First, Higuchi et al. reported GKRS fractionated with an interval [26]. The one-year local control rate was 75.9% in a study of 43 patients with large brain metastases (greater than 10 cu cm) who underwent staged GKRS of 30 Gy in three fractions every two weeks [26]. Later, Higuchi et al. also summarized the results of a reported series of two-staged and three-staged GKRS in the literature [6]. Local recurrence rates were 7-15%, and complication rates 1.9-6.4%. The aim underlying these staged-GKRS with an inter-fraction time of two-to-four weeks is to reduce the tumor size sufficiently so that the second treatment can be performed more safely on a smaller volume. Serizawa et al. compared the multi-institutional results of three- and two-staged GKRS for large brain metastases [13]. Large tumors, of 10.0 to 33.5 cu cm in volume, were treated with 9.0 to 11.0 Gy (single fraction dose) in three-stage GKRS and 11.8 to 14.2 Gy in two-stage GKRS. The incidence of serious radiation-related adverse events was 3.0% and 4.0%, respectively, with this difference not significant.

In this study, we showed the clinical results of one-day two-fraction GKRS applied to relatively large and not so large brain metastases (median PIV of 10.1 ml). One-day two-fraction GKRS has the merit of precise targeting with rigid fixation using a skull frame and provides an advantage by fractionation. The patient must be subjected to skull frame placement during one daytime period. Besides the brain metastasis cases described in this paper, we have also treated some cases with other diseases, including one retinal metastasis, four benign skull base tumors, and one arteriovenous malformation, in the same fashion during the same period. No acute adverse effects were observed in any of these cases (unpublished data). Indications of one-day two-fraction radiosurgery were large target volume and proximity of important radiation-vulnerable structures, including optic pathways and motor cortex. The optic pathways are important structures but vulnerable to radiation [29]. Milano et al. described the dose tolerance of the optic pathways in single- and multi-fraction SRS [29]. Inoue et al. reported the importance of 14 Gy equivalent dose-volume in hypofractionated CyberKnife SRS for metastases in the critical areas [30].

## Conclusions

A relatively high dose may be safely delivered to somewhat large lesions, those close to the important structures, or those with pre-existing perifocal edema by one-day two-fraction GKRS. Local control was good, except for one relapsed SCLC metastasis case. Evaluation in more cases with a longer follow-up period will be necessary to determine the definite indications and optimal prescription dose.
